# Low-Power, Multimodal Laser Micromachining of Materials for Applications in sub-5 µm Shadow Masks and sub-10 µm Interdigitated Electrodes (IDEs) Fabrication

**DOI:** 10.3390/mi11020178

**Published:** 2020-02-08

**Authors:** Cacie Hart, Swaminathan Rajaraman

**Affiliations:** 1 Department of Materials Science & Engineering, University of Central Florida, Orlando, FL 32816, USA; chart@knights.ucf.edu; 2 NanoScience Technology Center, University of Central Florida, Orlando, FL 32816, USA; 3 Department of Electrical & Computer Engineering, University of Central Florida, Orlando, FL 32816, USA; 4 Burnett School of Biomedical Sciences, University of Central Florida, Orlando, FL 32816, USA

**Keywords:** multimodal laser micromachining, ablation characteristics, shadow mask, interdigitated electrodes

## Abstract

Laser micromachining is a direct write microfabrication technology that has several advantages over traditional micro/nanofabrication techniques. In this paper, we present a comprehensive characterization of a QuikLaze 50ST2 multimodal laser micromachining tool by determining the ablation characteristics of six (6) different materials and demonstrating two applications. Both the thermodynamic theoretical and experimental ablation characteristics of stainless steel (SS) and aluminum are examined at 1064 nm, silicon and polydimethylsiloxane (PDMS) at 532 nm, and Kapton^®^ and polyethylene terephthalate at 355 nm. We found that the experimental data aligned well with the theoretical analysis. Additionally, two applications of this multimodal laser micromachining technology are demonstrated: shadow masking down to approximately 1.5 µm feature sizes and interdigitated electrode (IDE) fabrication down to 7 µm electrode gap width.

## 1. Introduction

In recent years, laser technology has been examined as an exciting material processing method for both industrial and academic researchers [1,2,3,4]. The high quality of laser beams allows for improved micromachining precision for a variety of materials [5,6]. Additionally, the compact size, high efficiency, cost-effectiveness, direct machining, 3D fabrication, and ease of integration are appealing to academic researchers with limited benchtop area and lean budgets.

Laser micromachining, specifically, involves the ablation of materials where the features produced by the laser are in the micro-scale [6,7]. Laser micromachining techniques are currently employed in the automobile [8], medical [2,9], semiconductor [10,11], and solar cell industries [12]. Lasers used in micromachining are available in a wide range of wavelengths (ultraviolet to infrared), pulse durations (micro- to femtosecond), and repetition rates (single pulse to megahertz) [6,8]. Because of this flexibility, laser micromachining allows for the processing of a variety of materials.

**Figure 1:**
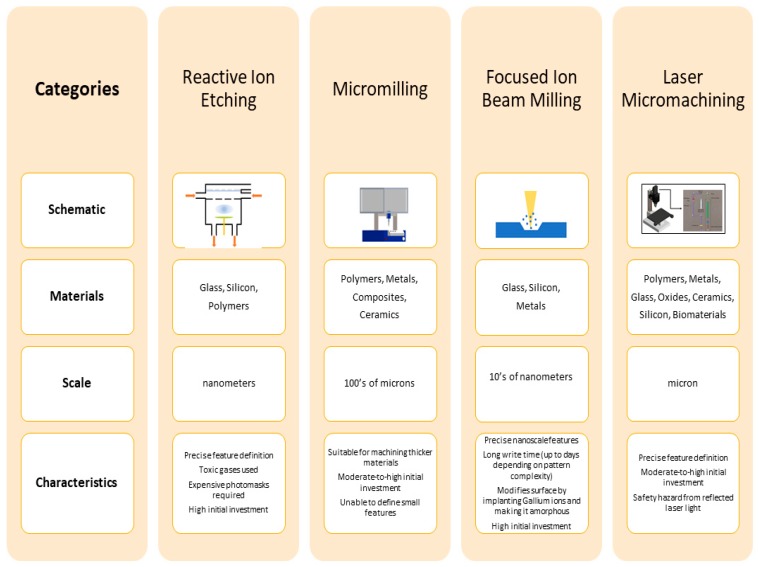
Comparison of three widely used direct-write fabrication methods (reactive ion etching (RIE) [15,17], micromilling [14], and focused ion beam (FIB) [13,16,18]) to laser micromachining. Each of these methods allows for high aspect ratio, serial, single-substrate fabrication. In the case of RIE multi-wafer/multi-substrate parallel processing is also possible as with some laser micromachining examples as well for higher throughput micro/nanofabrication.

Figure 1 compares laser micromachining to three commonly used direct-write microfabrication methods [13,14,15,16,17,18,19]. All these methods involve a mechanism for removing material directly from a substrate in a desired pattern using computer-controlled machinery. While other patterning methods, such as photolithography are often used, they are not direct-write techniques and involve several steps to pattern materials; thus, a comparison to these other methods is not provided in this figure. Photolithography is, of course, required for one of the methods depicted (reactive ion etching or RIE) for performing feature definition followed by RIE to “engrave’ the feature in the substrate beneath [15,16,17,19]. Since RIE requires photolithography, the process involves more steps when compared to the other methods depicted in Figure 1 [17]. Micromilling is a very technically simple process; however, this simplicity comes at the expense of frequent drill bit brakeage and the inability to produce features in the sub-100 µm range [14]. As with some RIE technologies, focused ion beam (FIB) milling can define nanoscale features; however, the write process can take days depending on the pattern complexity. Additionally, in FIB-based milling processes, the material surface may become amorphous due to the implantation of gallium ions [13,16,17,18]. The comparison depicted in Figure 1 is by no means comprehensive and has been included to show the characteristics of laser-based micromachining with some other commonly used technologies for patterning materials in micromachining and micro-electro-mechanical systems (MEMS). Several other techniques for direct subtractive fabrication of micromachined features are available such as electrical discharge machining (EDM) [20], ultrasonic drilling [21], water jet cutting [22] etc.

Laser micromachining has occasionally been used for shadow mask fabrication; however, the state of the art in laser micromachining (minimum feature size of 10 µm) [23] (to date) has relied entirely on single-wavelength excimer, CO_2_, and Nd:YAG (neodymium-doped yttrium aluminum garnet) lasers [1,3,12,23,24,25,26,27,28,29,30,31,32,33,34,35,36]. To the best of our knowledge, multimodal laser micromachining has not been used for shadow mask fabrication. While the term “multimodal” in the laser field typically means that the laser contains multiple transverse electromagnetic modes, the laser micromachining tool used in this work has the ability to operate at several wavelengths in the same tool, a rare capability not afforded by many lasers. Specifically, with respect to shadow mask microfabrication with lasers, several impressive research efforts are reported in literature: Klank, et. al. fabricated 85 µm channels in polymethyl methacrylate (PMMA) with a CO_2_ laser [1]. Fan, et. al. also used a CO_2_ laser to micromachine 250 µm lines in wax for use as a shadow mask [3]. Tahir et. al. used a 1064 nm Nd:YAG laser to fabricate 250 µm channels in wood, glass, plastic, and rubber [12]. Chung, et. al. fabricated shadow masks with minimum feature sizes of 200 µm with a 785 nm Ti: sapphire laser [26]. Shiu, et. al. machined 140 µm channels in low-carbon steel for use as a shadow mask using an excimer laser [34].

The lasers used in the aforementioned research can produce shadow masks for MEMS applications, but they are unable to micromachine highly precise features on the scale of a single micron, unless they operate in the femtosecond regime [3,7,23,24,30,37]. The higher power of these lasers allows for the processing of thicker materials in a more reasonable time scale; however, this capability comes at the price of benchtop machining, high costs, space, high power usage (GW), and absence of multimodality to micromachine several materials with the same tool. Characterization of lasers used for materials processing is necessary for the proper use of such tools. By understanding the necessary energy, repetition rate/frequency, spot size, and depths that can be micromachined with various processing conditions in specific materials, users can subsequently optimize their experiments for successful results in different applications.

In this paper, we report the full characterization of a multimodal laser micromachining tool and two applications of the usage of microstructures fabricated using the tool. Multimodal laser micromachining allows for a wide range of materials processing capabilities; however, this comes at the price of limited power and nanosecond ablation. These tools allow users to operate at specific laser wavelengths (1064 nm, 532 nm, and 355 nm, in our case) and reduced powers (2.6 mJ maximum power). These conditions, however, provide greater control over the material selectivity for laser micromachining and ablation depths. Specifically, one can remove a material from a device while leaving the remainder of the constituent materials unaffected [38].

Typically, such laser micromachining tools are employed in liquid crystal display (LCD) repair, semiconductor failure analysis, and removing shorts and passivation layers in integrated circuits [6,39]. In this paper, we characterize the multimodal tool for the laser micromachining of six (6) different materials: Stainless steel (SS) and aluminum, to be machined with infrared (IR) mode, polyethylene terephthalate (PET) and Kapton^®^, to be machined with ultraviolet (UV) mode, and polydimethylsiloxane (PDMS) and silicon, to be machined with green mode. Subsequent shadow mask patterning allows for the definition of organic and inorganic layers and the development of a fully functional interdigitated electrode (IDE) devices. These devices are further characterized in this paper.

## 2. Microfabrication Method Overview

**Figure 2:**
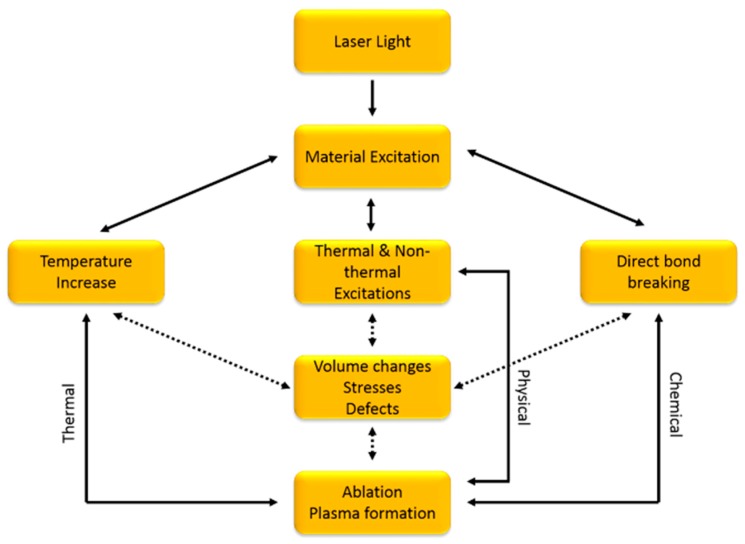
Mechanisms of ablation in laser micromachining. Substrate removal can be a result of thermal ablation, physical ablation, chemical ablation, or a combination of these mechanisms.

In laser micromachining, the laser beam is collimated into a small spot and patterning is achieved by either moving the substrate within a fixed beam or rastering the laser across a surface [6]. The desired machining patterns simply need to be drawn in a CAD program (such as AutoCAD, Autodesk, San Lafayette, CA, USA) and imported as a drawing exchange format (DXF) file into the control program of the laser micromachining tool. Once the program is executed with the laser, substrate material removal can be a result of photochemical, photothermal, or photophysical ablation [40], as shown in Figure 2. Commonly used processes include laser cutting, scribing, drilling, or etching to produce relief structures or holes on a substrate in ambient temperatures [3,8,23,27,40,41,42]. The power of this technique lies in the ability to construct desired patterns on arbitrarily shaped surfaces, with the only limitation being the degrees of freedom and the resolution of the motion controller. Laser micromachining is considered a rapid prototyping technique because designs can be changed immediately without the need to fabricate new molds or masks.

**Figure 3:**
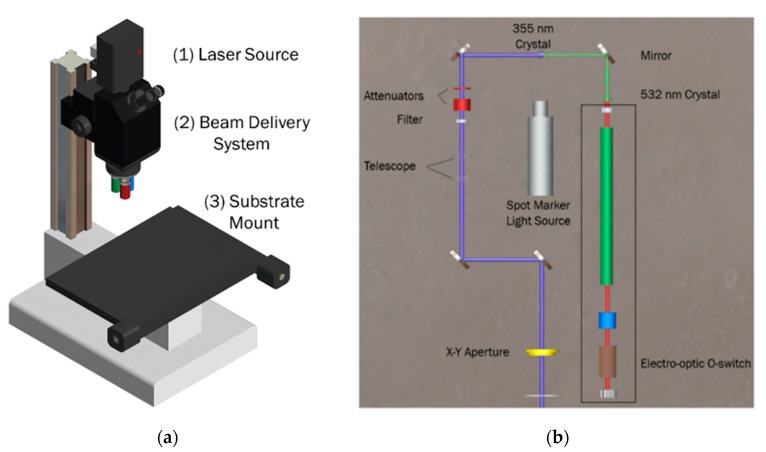
(**a**) External schematic of a QuikLaze 50ST2 Multimodal Laser Micromachining System. The three components of every laser micromachining system are shown: (1) the laser source box; (2) the beam delivery system; and (3) the motorized substrate mount. (**b**) Internal schematics of the laser source box. This shows the three crystals and a switch that create the different wavelengths of light the system produces with the ability to switch between three wavelengths.

Every laser micromachining system is comprised of three parts, as shown in Figure 3a for the multimodal laser micromachining tool: (1) the source laser, (2) the beam delivery system, and (3) the substrate mounting stage. Obviously, the laser source is at the heart of the system, as it determines which substrates and feature sizes can be micromachined [8]. The system used in this work has a multimodal laser source that allows for switching between three wavelengths of light: 1064 nm infrared (IR mode), 532 nm visible green (Visible mode), and 355 nm ultra-violet (UV mode). This wavelength switching allows for greater substrate compatibility and a wide array of feature sizes in an extremely compact benchtop system.

Beam delivery involves optical components, including fixed focusing objects and mirrors, galvanometric scanners, optical fibers, wave-guides, apertures, and q-switches, that are used to generate the laser spot. The selection of these optical components depends on the working distance, desired spot size, and required energy [6,8,41,43]. The combination of the laser source and optical components of the beam delivery system determines the ultimate properties of the laser beam. The beam delivery system used in this work is depicted in Figure 3b with greater detail.

Lastly, the substrate mounting system depends on how the rastering occurs on the tool. If the laser beam is to be rastered over the substrate surface, then a stationary substrate mount may be used. However, in most cases, including the system used in this work, it is more desirable to raster the substrate itself within a laser beam. In this instance, the substrate mount is manipulatable in the x- and y-directions, and even in the z-direction in some cases.

In the multimodal laser system used in this work, the laser beam is initially generated as a 1064 nm IR mode, shown in Figure 3b. The IR laser beam is subsequently passed through two crystals in the beam delivery system that produce the two additional wavelengths. The beam delivery system employs filters prior to the microscope optics to filter out the unnecessary wavelengths, allowing only the desired wavelength to interact with the substrate surface.

## 3. Theoretical Background

A thermodynamic approach to calculating the theoretical “depth of cut” for the materials tested in this work was adapted from Schütz, et. al. [10]. This formula is based solely on material properties and laser energy. Examining the depth of cut in this manner allows for a more fundamental understanding of the interaction between the laser and the material with fewer assumptions when compared to a molecular approach used for photochemical ablation [10,42,44].

(1) $$a_{p}=\frac{\Phi }{\rho \int_{T_{room}}^{T_{v}}c_{p}\left(T\right)dT+\sum_{i=1}^{n}H_{ph}^{v}}-d$$

**Figure 4:**
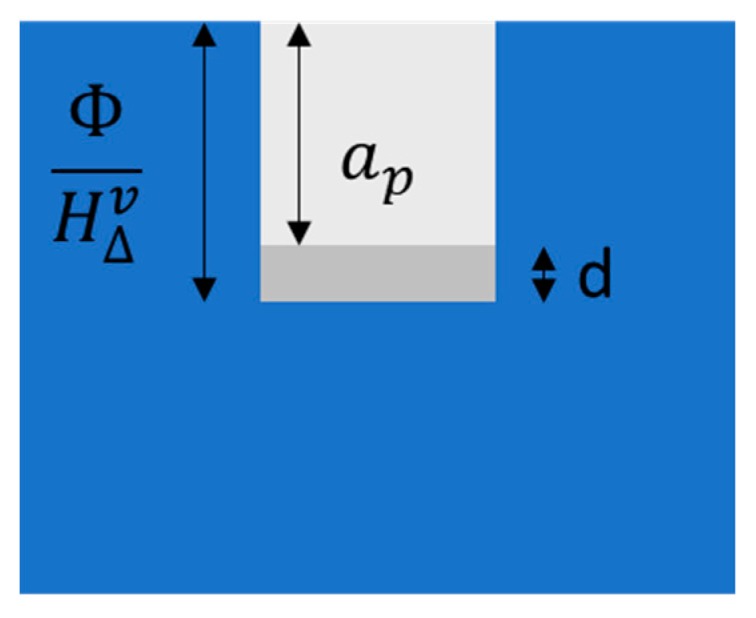
Schematic showing the variables in the thermodynamic theory for evaluation of the depth of cut.

Equation (1) shows the relationship between the depth of cut per pulse (*a_p_*), the laser fluence (*Φ*), and involved material properties, including density (*ρ*), vaporization temperature (*T_v_*), heat capacity (*c_p_*), and the phase change enthalpy (*H^v^_ph_*). The d term (right-hand side of the equation) is a correction term that includes optical and thermal losses. This equation represented schematically (Figure 4) balances intrinsic energy and enthalpy, which are state variables and omits fundamental process parameters that are not state variables (i.e., particle dynamics) [45,46]. While the inclusion of these fundamental process parameters could lead to a more accurate theoretical solution, the complexity would be vastly increased due to the use of non-linear partial differential equations and the need for sophisticated simulation software to solve such equations [5,6,10,40,44,47]. Additionally, since this laser micromachining setup does not operate in the femtosecond regime, ablation occurs through melt expulsion and redeposition driven by the vapor pressure and the recoil pressure of light [44,47,48]. As a result, a simple thermodynamic analysis was performed to extract the relationship between the “depth of cut” and laser fluence which can be compared to the results obtained through experiments.

## 4. Materials and Methods

### 4.1. Multimodal Laser

A QuikLaze 50ST2 multimodal laser (New Wave Research Inc., Fremont, CA, USA) was used for all the laser micromachining performed in this paper. A selection of three wavelengths as mentioned in the previous sections allows the laser to be tailored to a specific application. The microscope of the laser system is equipped with 10×, 50×, and 100× lenses, each with specific wavelength limitations. Because of additional filters in the microscope lenses, the green wavelength can be used through any of the lenses, while the UV and the IR can only be used through the 50× and 100× lenses, respectively.

The laser outputs a 5 mm diameter Gaussian beam, which is then shaped into a rectangle by the XY aperture. The size of this rectangle is determined by user inputs into the control software. The maximum pulse duration of the laser is 5 ns for all wavelengths; however, it can be adjusted by the user in the program. Additionally, the laser fluence depends on the user specifications in the control software and the wavelength of light used, as the laser output energy can be adjusted by the user. The fluence ranges from a maximum of 27,000 J/cm^2^ to a minimum of 1.08 J/cm^2^.

### 4.2. Materials Used

To ensure a comprehensive study of multimodal laser micromachining, several materials were machined. Success in laser micromachining for a given material is typically determined only by the choice of wavelength because each material reacts differently to a specific wavelength. In general, metals absorb shorter wavelengths more effectively than longer wavelengths [6,8]; however, there are limitations to how effectively material is removed at shorter wavelengths determined by the microscope optics used. Because the amount of light transmitted through the microscope objectives required for each wavelength varies, the “effective” absorption of the material at a given wavelength is changed.

Stainless steel 12.5 µm thick (type 304) (Trinity Brand Industries, Countryside, IL, USA) and 16 µm-thick aluminum foil (Reynolds Group Holdings, Auckland, NZ, USA) were machined using the 1064 nm IR wavelength through the 100× microscope lens. The absorbances of these materials at this wavelength is 37% and 5%, respectively [42]. Kapton^®^ of thickness 12.5 µm (DuPont, Wilmington, DE, USA) and 25 µm-thick polyethylene terephthalate (PET) (McMaster-Carr, Elmhurst, IL, USA) were machined using the 355 nm UV wavelength through the 50× microscope lens. The absorbances of these materials at this wavelength are 22.5% and 12%, respectively [40,43]. Finally, Silicon (University Wafer, Boston, MA, USA) and polydimethylsiloxane (PDMS) (Dow Corning, Midland, MI, USA) were micromachined using the 532 nm green wavelength through the 10× microscope lens. The absorbances for these materials at this wavelength are 25% and 72%, respectively [10,12,49].

### 4.3. Laser Characterization

**Figure 5:**
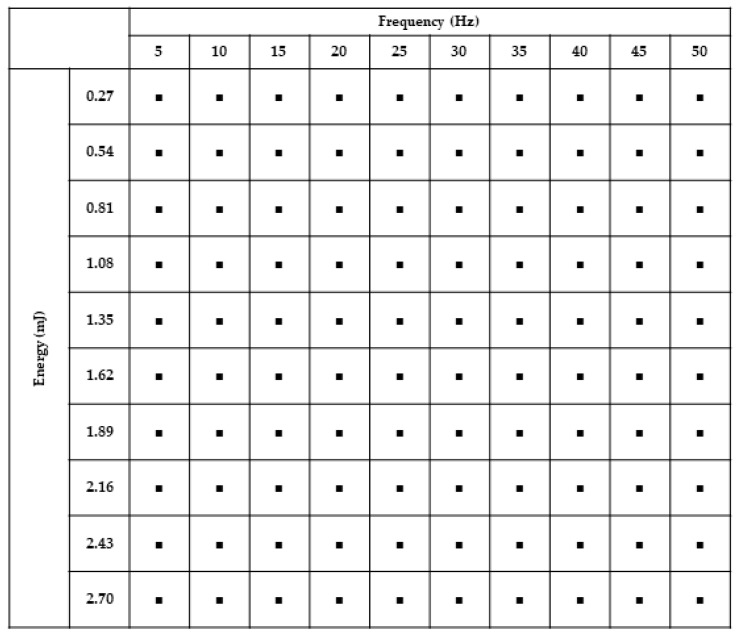
Layout of the grid of spots laser cut into the materials. The grid was designed to have 100 µm distances between the spots.

To establish protocols for processing each material, characterization grids were machined for all six (6) materials. These grids consisted of 100 spots, as shown schematically in Figure 5, and were designed in SolidWorks (Dassault Systems, Waltham, MA, USA).

DXF pattern files were subsequently uploaded into the New Wave Laser program, and each spot was assigned a specific frequency (range of 5 Hz to 50 Hz) increasing by 5 Hz increments along the x-axis of the grid, and a specific energy from 10% (0.27 mJ) to 100% (2.7 mJ) increasing along the negative y-axis of the grid by 10%. The laser spot size was varied for each grid in order to provide full characterization of the laser’s capabilities over a wide range of fluence. Grids were subsequently patterned in all 6 materials using the multimodal laser.

All grids were imaged using scanning electron microscopy (JEOL JSM-6480, Tokyo, Japan). Full images of the grids were obtained, as well as images of the individual spots in both flat and 45° angled orientations. ImageJ (NIH, Bethesda, MD, USA) was used to characterize the depth of the laser cut, as well as the resultant spot size.

### 4.4. Shadow Masks

Shadow masks for the patterning of materials were fabricated from Kapton^®^, SS, PET, and aluminum substrates. These materials were chosen because they are all commonly available in most microfabrication laboratories and it was determined that they could be ablated all the way through the material using the multimodal laser, a necessity for shadow masks. Full coverage, circle-on-line IDE shadow masks were designed using SolidWorks and machined using the multimodal laser and the appropriate wavelength for the substrate material. The shadow masks were then imaged using the scanning electron microscope (SEM, JEOL JSM-6480, Tokyo, Japan) to characterize the design (CAD dimensions) to device (fabricated shadow mask) translation. These shadow masks were subsequently used to pattern both metal and gelatin for design to device studies, as described in Section 4.5.

Additionally, traditional interwoven comb IDE shadow masks were fabricated to test the lowest feature size limits of the multimodal laser. These structures allow for more rapid shadow mask fabrication at the lowest possible widths ensuring higher sensitivity in IDE assays; thus, allowing for any necessary adjustments to achieve the desired electrode gap width. Due to the size of these structures, atomic force microscopy (AFM) (Anasys Instruments, Santa Barbara, CA, USA) and SEM (JEOL, Tokyo, Japan) were used to characterize the electrode gap widths of these shadow masks.

Lastly, because of the widespread use of PDMS in microfluidics, several lines of 2 mm length were laser micromachined into this material in order to determine the depth of cut for a given number of laser passes. All lines were micromachined through the IR microscope lens with the green laser at 2.7 mJ with an X-Y aperture area of 50 µm^2^. The number of laser passes was varied from 5 to 40 passes. In this mode of operation, the laser beam is continuously scanned across the surface of the material in the desired pattern.

**Figure 6:**
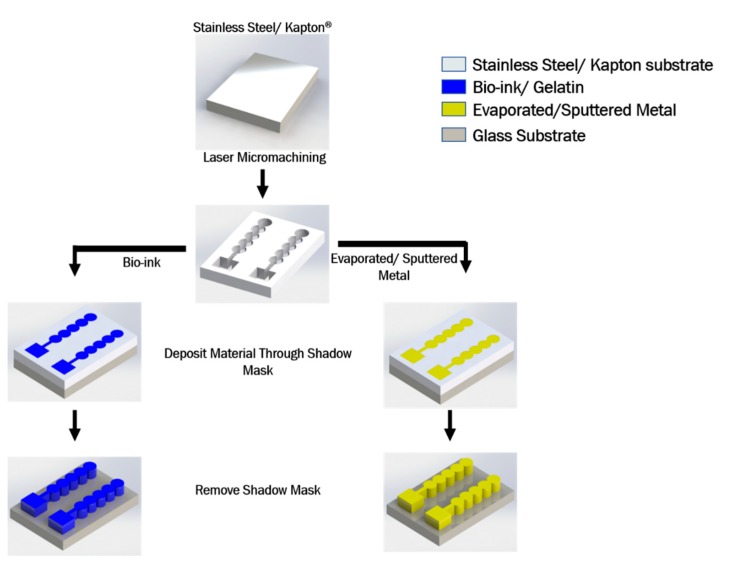
Schematic of laser micromachined shadow mask fabrication and subsequent materials patterning demonstration.

The process flow for the shadow mask fabrication and subsequent patterning of organic and inorganic layers is depicted in Figure 6.

### 4.5. Patterning of Organic and Inorganic Layers

In order to access the accuracy of low power, multimodal laser micromachining, material patterning through multimodal laser micromachined shadow masks was performed.

#### 4.5.1. Metal Patterning

The Kapton^®^, SS, aluminum, and PET multimodal laser micromachined shadow masks were affixed onto glass microscope slides (Fisher Scientific, Hampton, NH) with Kapton^®^ tape (DuPont, Wilmington, DE, USA). Titanium-Gold (Ted Pella, Redding, CA, USA) electrodes, traces, and contact pads of 5nm-30 nm thickness respectively were subsequently deposited (Deposition rate: 1 Å/s at 1 × 10^−6^ Torr) through these shadow masks onto glass microscope slides via electron beam evaporation (Thermionics, Port Townsend, WA, USA). After metal patterning, the shadow masks were removed by carefully detaching the Kapton^®^ tape from the glass slide with tweezers. Transmitted light microscopy (TS2 Inverted Microscope, Nikon, Tokyo, Japan) was used to image the patterned metal structures. ImageJ (NIH, Bethesda, MD) was used for further optical analysis of the captured images and measurement of the electrode structures.

#### 4.5.2. Gelatin Patterning

Gelatin (Millipore Sigma, St. Louis, MO, USA) and deionized (DI) water were mixed to make a 5 wt % gelatin solution to act as a sample bioink. Kapton^®^ IDE shadow masks were dipped in DI water to allow for better adherence to the glass microscope slide substrates. The 5 wt % gelatin solution was subsequently pipetted onto the glass slides through the shadow masks. The gelatin solution was allowed to set, then the shadow masks were carefully removed to expose the gelatin IDE patterns. The resultant gelatin structures were imaged using a Nikon TS2 inverted microscope.

### 4.6. Impedance Characterization of Metal Patterns

The full spectrum (10 Hz to 100 kHz) impedance of each of the resultant patterned metal IDE structures was measured with a BODE 100 impedance measurement system (Omicron Labs, Klaus, Austria). Dulbecco’s phosphate buffered saline (DPBS) (Gibco, Waltham, MA, USA) acted as the electrolyte solution and platinum-titanium wire (eDAQ, Deniston East, NSW, Australia) was used as a counter electrode used during the impedance measurements. Impedance data was extracted and plotted with Origin 2016 (OriginLab, North Hampton, MA, USA).

## 5. Results and Discussion

### 5.1. Laser Characterization

**Figure 7:**
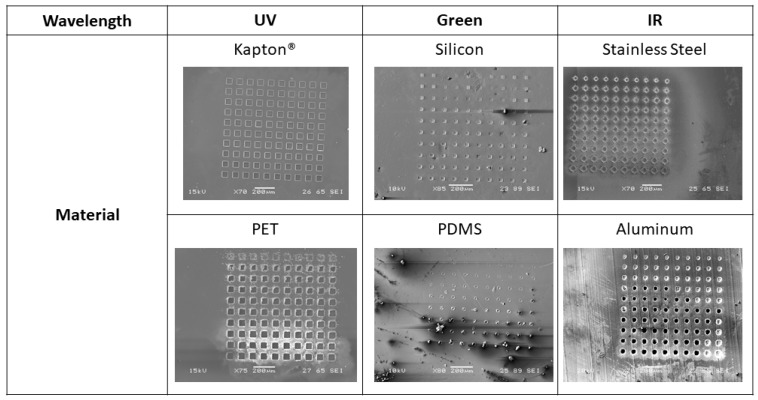
Scanning electron microscope (SEM) images of the laser characterization grids for each of the six materials. Kapton^®^, SS, polyethylene terephthalate (PET), and aluminum are all at the maximum spot size for their respective wavelengths. Silicon and polydimethylsiloxane (PDMS) are both at 50% spot size to show the grid in its entirety. All scale bars are 200 µm.

Laser characterization grids were fabricated for each of the six materials for a range of spot sizes as described in the Materials and Methods section. The spot sizes for the materials depended on the microscope objective that was used for the ablation. The spot sizes ranged from 50 µm down to 2 µm for IR ablation through the 100× lens for SS and aluminum, 60 µm down to 4 µm for UV ablation through the 50× lens for Kapton^®^ and PET, and 250 µm down to 20 µm for green ablation though the 10× lens for silicon and PDMS. SEM imaging of these grid structures, and subsequent processing in ImageJ was used to calculate the depth of cut for each spot micromachined by the laser. Figure 7 shows examples of the characterization grids for each of the six materials at the maximum spot size for each wavelength. For PET, Kapton^®^, SS, and aluminum, full ablation (through vias, all the way through the substrate) is achieved for the full range of power and frequency combinations shown in Figure 5. Since these materials were fully ablated within the 10 pulses used, the ablation depth was averaged over the number of spots. Some deviation from the theoretical calculation for these materials was observed. Ablation rates of 46.3 µm/mJ for Kapton^®^ and SS, 92.6 µm/mJ for PET, and 59.3 µm/mJ for aluminum were achieved. Neither silicon nor PDMS were able to be fully ablated through for any power and frequency combination. Silicon began measurable ablation at a minimum of 35 Hz and 2.43 mJ and had an ablation rate of 1.4 µm/mJ. PDMS began measurable ablation at a minimum of 35 Hz and 2.16 mJ, which gives an ablation rate of 1.5 µm/mJ.

**Figure 8:**
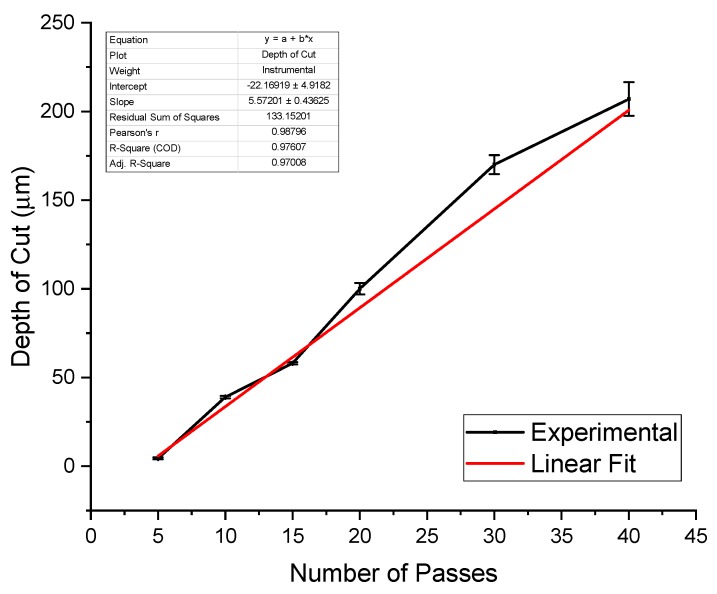
Results from scribing lines in PDMS with a varied number of laser passes. The depth of cut increases linearly with the number of laser passes as expected. This allows for the direct tuning of microchannel depth by selecting the number of passes the laser makes in order to cut PDMS.

Traces (lines) were scribed in PDMS using the green laser through the IR lens with between 5 and 40 passes and a 50 µm laser spot size. Cross-sectioning the scribed lines carefully using a razor blade, followed by SEM imaging of the cross section, resulted in the measurement of the depth of cut for each number of laser passes. Figure 8 shows the linear relationship that was found between the depth of cut and the number of laser micromachining passes. Characterizing this relationship allows for the fabrication of microchannels of specific depths in PDMS by varying the number of laser passes.

#### Comparison to Theoretical Values

**Figure 9:**
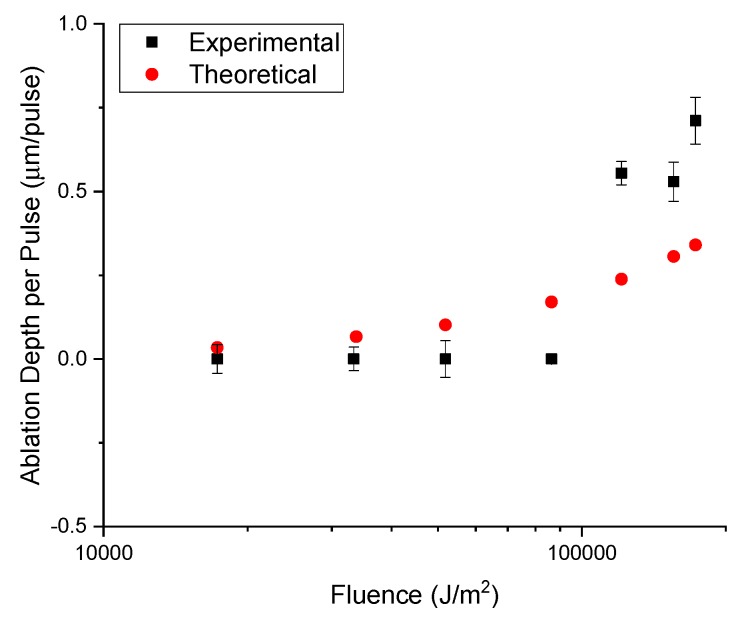
Comparison of theoretical and experimental ablation depth for a range of fluences in silicon.

Figure 9 and Table 1 show the comparison between the theoretical ablation depth model and our experimental results. For silicon and PDMS, the thermodynamic model fits well. The comparison in ablation depth per pulse for both materials between theory and practice is in the same order of magnitude. Furthermore, the ablation depth per pulse/ depth of cut plots for both materials show a clear correlation between the experimental and theoretical data. Neither one of these materials are ablated all the way through by the laser.

Conversely, deviation from theory is observed in the experimental ablation depth per pulse comparison for Kapton^®^, SS, PET, and aluminum. Kapton^®^ and SS perform similar to silicon and PDMS as observed in Table 1 with both the theoretical and experimental values in the same order of magnitude with experimental values being smaller than theoretical values. The discrepancy between PET and aluminum is roughly an order of magnitude higher predicted theoretical values. The laser ablated through these materials at some point (difficult to measure experimentally in our current setup), so deviation is expected in all these materials. For example, both Kapton^®^ and SS could have shown an average ablation depth of ~3 µm per pulse, and both PET and aluminum could have shown an average ablation depth of approximately ~16 µm per pulse. Because of the thickness of these materials, when the normalization of the ablation depth to the number of pulses is performed, the experimental values are determined to be substantially lower. The key results from the experimental data in this case is that the laser is capable of fully ablating these thicknesses and that a reasonable number of pulses can be used to ablate even thicker samples of these materials. These results also show the limitations of a thermodynamic approach, as the material thickness is not considered in the calculations.

### 5.2. Applications

Two applications of this multimodal laser micromachining technique were additionally demonstrated in this work namely the microfabrication of shadow masks and IDEs.

#### 5.2.1. Shadow Masks

Shadow masking technology is an integral part of fabricating micro/nanostructures for prototyping in microelectronics, optical, microfluidic, MEMS, packaging, and biomedical lab-on-a-chip applications [11,23]. Typical methods for producing shadow masks, such as photolithography and deep reactive ion etching (DRIE) or ion beam milling, are expensive, require cleanroom-based fabrication, expensive vacuum equipment, ultra-pure air filtration, and advanced know-how [3,50]. Multimodal laser micromachining, on the other hand, is simple, cost effective, and makerspace-compatible, all vital attributes for cell-based assays and microfluidics applications.

**Figure 10:**
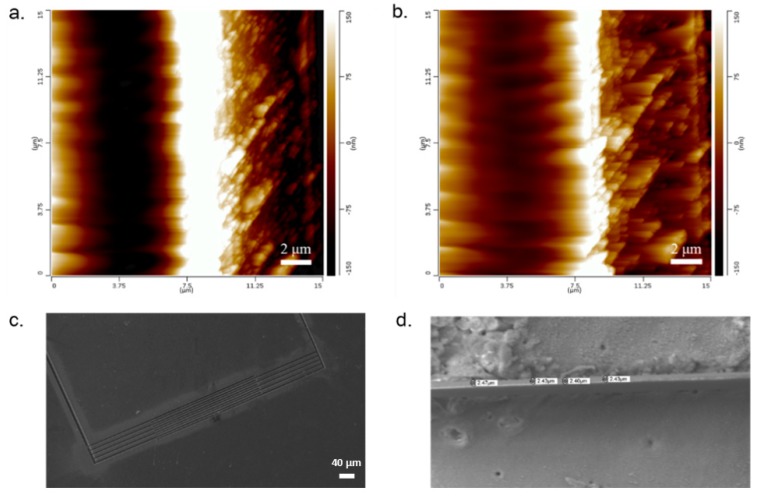
Images of sub-5 µm trace widths in Kapton^®^. (**a**) Atomic force microscopy (AFM) image of 3.5 µm trace width (white area). (**b**) AFM image of 1.5 µm trace width (white area). (**c**) SEM image of full comb finger electrode structure (approx. 5 µm trace width). (**d**) SEM image of shadow mask feature of approximately 2.43 µm trace width.

Fabrication of the shadow masks down to ~1.5 μm was successfully demonstrated, as shown in Figure 10. To the best of our knowledge, this is the lowest feature size demonstrated for laser defined shadow masks [3,23]. Previous work with laser micromachining has produced feature sizes down to ~10 μm. As a result, patterns that are an almost an order of magnitude better than the state of the art are reported in this paper.

#### 5.2.2. Patterning Through Shadow Masks

The laser micromachined shadow masks were further used to pattern both metal and gelatin/bio-ink as described in Section 4.5. The organic and inorganic layer patterning can be utilized for applications such as accurate cell placement in single cell and culture assays, precision confinement and growth of cellular constructs, tissue engineering, metal micro/nanoelectrodes, definition of organic insulation layers, and other lab-on-a-chip and diagnostic applications [3,23,41,49,51].

**Figure 11:**
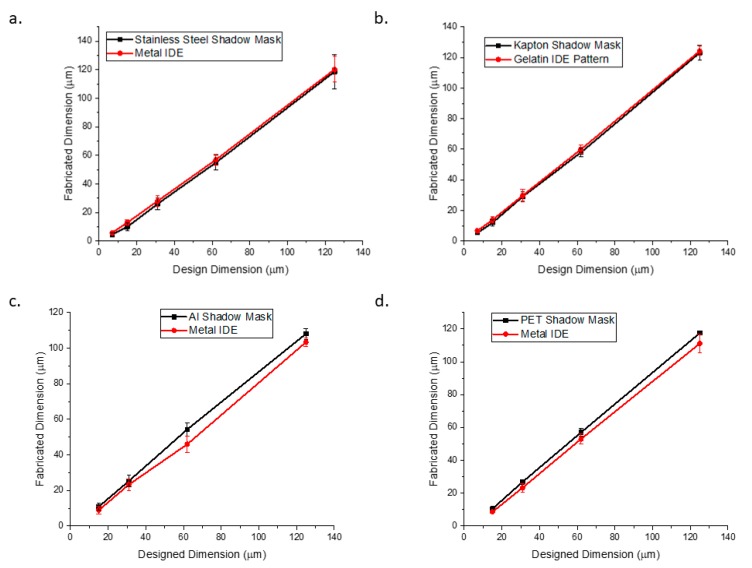
Design to shadow mask to device (interdigitated electrode (IDE) on glass) for the four materials that cut all the way through the substrate: (**a**) stainless steel to Ti-Au metal IDE; (**b**) Kapton^®^ to gelatin IDE pattern; (**c**) aluminum to Ti-Au metal IDE, and (**d**) PET to Ti-Au metal IDE.

Design of a shadow mask to microfabricated device translation for the four materials that the multimodal laser was completely able to micromachine in its entirety are shown in Figure 11. Aluminum, PET and SS shadow masks were used to fabricate metal IDEs while Kapton shadow masks were used for fabricating a gelatin/bio-ink IDE. It was observed that Kapton^®^ and SS demonstrated the best design to device translation for 125 µm to 7 µm. Both SS and Kapton^®^ showed minimal thermal damage from laser microfabrication at their respective laser wavelengths (1064 nm and 355 nm). Additionally, both materials have coefficients of thermal expansion (both approximately 20 × 10^−6^ K^−1^) which are 2× larger than the thermal expansion of glass (9 × 10^−6^ K^−1^) [46] theoretically suggesting better translation results and experimentally verified in Figure 11 (for N = 3 measurements at the various design values). The best design to device translation was observed to be ~98% for the 7 µm gelatin features due to the rapid deposition and curing of the gelatin. The worst design to device translation for SS was observed to be 95% for 125 µm metal IDE on glass due to the higher run-off possibilities during the electron beam evaporation process [5]. PET and aluminum demonstrated a deviation from the designed IDE pitch by 11.2% and 25.8% at maximum pitch, respectively. PET has a coefficient of thermal expansion (80 × 10^−6^ K^−1^) [46] that is nearly an order of magnitude larger than that of glass, so some deviation from the design dimensions is expected due to thermal mismatch effects during e-beam evaporation. The aluminum shadow mask exhibited physical melting during the laser micromachining process, which could explain the large deviation from design dimensions.

**Figure 12:**
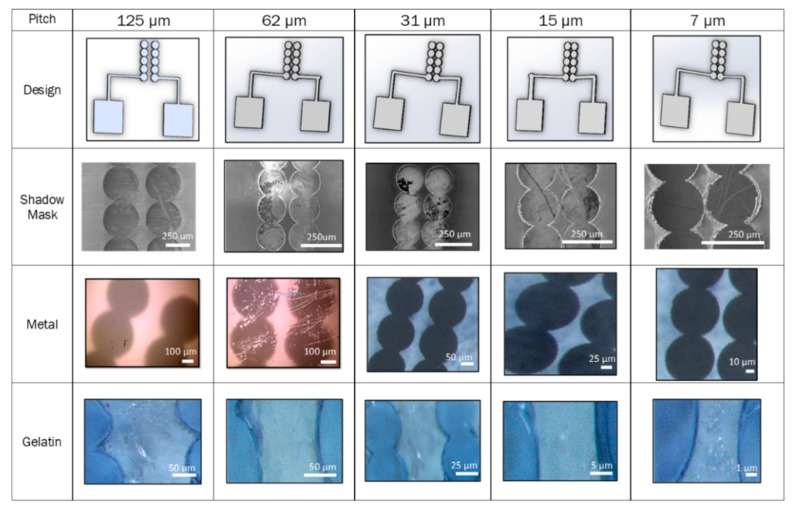
Results of laser micromachining of the shadow masks, as well as inorganic and organic layer patterning. The design of an interdigitated electrode (IDE) was translated into metal (titanium-gold) and bioink (gelatin).

Figure 12 shows the design schematic, SEM images of the shadow masks, and transmitted light microscope images of both Ti-Au metal patterning and gelatin bioink patterning for circular fill IDE patterns with electrode gaps from 125 µm to 7 µm. Metal patterning appeared to work best with Kapton^®^ or SS shadow masks because their coefficients of thermal expansion are closer to that of glass. Kapton^®^ and PET shadow masks worked best for gelatin patterning. Both shadow mask materials allowed for simple adhesion to the glass substrate utilizing surface tension effects by simply dipping the mask in DI water prior to attachment on glass substrates. Allowing the gelatin to fully set prior to shadow mask removal was key in preventing the patterns from bleeding.

**Figure 13:**
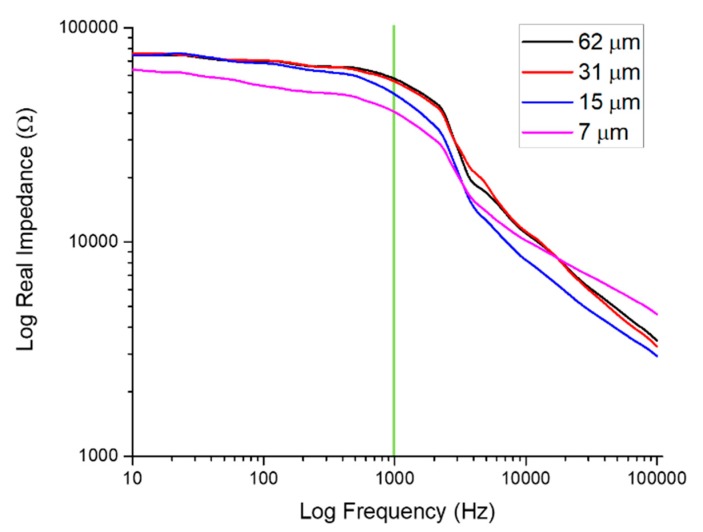
Full spectrum impedance measurements of several IDEs of varying pitch. The electrophysiologically significant frequency of 1 kHz (green line) reports impedances from approximately 39 kΩ (7 μm) to 56 kΩ (62 μm).

The impedance of the metallized IDEs (Figure 13) was found to decrease with decreasing electrode gap width as is expected [52] with the 1 kHz impedance decreasing by 33.47% between the extremities of the designs tested. Table 2 further illustrates impedances at key frequencies, clearly depicting resistive behavior at the lower and upper ends of the spectra and capacitive behavior in the mid-band with increasing impedance values as expected [52].

## 6. Conclusions

Complete characterization of the laser micromachining processes for six (6) commonly used microfabrication materials was developed in this work using a multimodal laser micromachining tool. Characterization of the QuikLaze 50ST2 multimodal laser for the laser micromachining of six (6) different materials demonstrated that the ablation depths that were experimentally obtained fit relatively well with a simple thermodynamic theory for most of the materials. While more complex theories or analysis [47] could improve the discrepancy, this technique is accurate enough to allow one to readily calculate possible ablation depths of a new material using such a laser micromachining tool. Additionally, two applications of multimodal laser micromachining were demonstrated: shadow mask fabrication and patterning of organic and inorganic materials in the sub-5 µm range and IDE fabrication in the sub-10 µm range. The ability of such a technique to allow for rapid prototyping of shadow masks and devices, combined with the compact, benchtop-friendly design gives multimodal laser micromachining tremendous promise as an efficient fabrication method in academic and industrial research settings.

## Figures and Tables

**Table 1:** Comparison of theoretical and experimental ablation depths.

Material	Thickness (µm)	Maximum Theoretical Ablation Depth Per Pulse (µm/pulse)	Experimental Ablation Depth per Pulse (µm/pulse)	Ablated Through After 10 Pulses?
Kapton	12.5	2.65	1.25 *	Yes
Stainless Steel	12.5	3.18	1.25 *	Yes
PET (polyethylene terephthalate)	25	15.6	2.5 *	Yes
Aluminum	16.3	16.3	1.63 *	Yes
Silicon	500	0.34024	0.7112	No
PDMS (polydimethylsiloxane)	100	0.25783	0.6481	No

* Limited due to full ablation of materials in less than 10 pulses.

**Table 2:** Values of the real part of the impedance at significant frequencies.

Impedance at Significant Frequencies	Frequency	Real Impedance (kΩ)				
		10 Hz	100 Hz	1 kHz	10 kHz	100 kHz
**Electrode Gap (μm)**	**7**	63.76	53.50	40.40	10.17	4.60
	**15**	74.21	68.37	48.91	8.24	2.90
	**32**	76.06	70.29	56.06	11.19	3.26
	**62**	74.77	70.05	57.74	10.99	3.46
		Resistive	Capacitive	Capacitive	Resistive	Resistive
